# Chest Wall Reconstruction of an Infected Open Costal Arch Fracture

**DOI:** 10.1016/j.atssr.2026.02.007

**Published:** 2026-02-24

**Authors:** Mikenzie Sturdevant, Adam Weiner, Shervin Eskandari, Dev Shah, Nicole Ivan, Imad Radi, Erin Switzer, Daniel Miller

**Affiliations:** 1 Department of Surgery, Medical College of Georgia, Augusta University, Augusta, Georgia; 2 Medical College of Georgia, Augusta University, Augusta, Georgia

## Abstract

A 55-year-old man presented with sepsis, 2 weeks after blunt chest trauma, with an open costal arch fracture. Imaging showed an extensive chest wall soft tissue infection. After resuscitation, soft tissue debridement was performed, and broad-spectrum antibiotics were started. Multiple debridements of the soft tissue were required in addition to allograft skin grafting. Once the soft tissue infection was under control, the costal arch fracture involving the costal cartilages was debrided. Chest wall reconstruction was performed successfully with bioabsorbable bars, an omental flap, and skin grafting. Long-term follow-up showed excellent viable tissue and no flail chest.

Chest wall reconstruction may be required for traumatic injuries, but associated infection related to trauma is a less common indication. The principles of chest wall reconstruction are to recreate chest wall rigidity, preserve chest wall mechanics, protect intrathoracic organs, and minimize deformity.^1^ The type of material used depends on the cause and the condition of the surrounding tissue. The material needs to reestablish rigidity and prevent paradoxical motion.^1^ Unfortunately, implantation of foreign material has an inherent risk of infection. In cases of infectious origin, biomaterials are superior in preventing hardware infection.^2^^,^^3^ Biomaterial prostheses, such as acellular dermal matrix, are used for temporary scaffolding to maintain chest wall integrity. Biologic meshes provide notable resistance to infection, exhibit biocompatibility, and may be retained even when residual infection persists.^4^

Necrotizing soft tissue infections (NSTIs) require extensive debridement of soft tissue as a result of rapid, life-threatening tissue necrosis.^5^ These infections lead to significant morbidity and mortality if they are not treated promptly and aggressively. Anterior chest wall NSTIs are rare. Despite advances in critical care, NSTIs still have high mortality rates, and any delay in treatment can worsen outcomes.^6^ This case involves a patient with a complex anterior chest wall NSTI who required multiple debridements and biomaterial reconstruction of an open costal arch fracture.

A 55-year-old man presented to the emergency department with progressive swelling, erythema, and purulent drainage from the lower anterior chest wall after a blunt chest trauma fall at home 2 weeks earlier (Figure 1A). A computed tomographic (CT) scan revealed extensive chest wall soft tissue infection (Figure 1B) arising from an open left costal arch fracture of avulsed seventh and eighth costal cartilages from the sternum.

**Figure 1:**
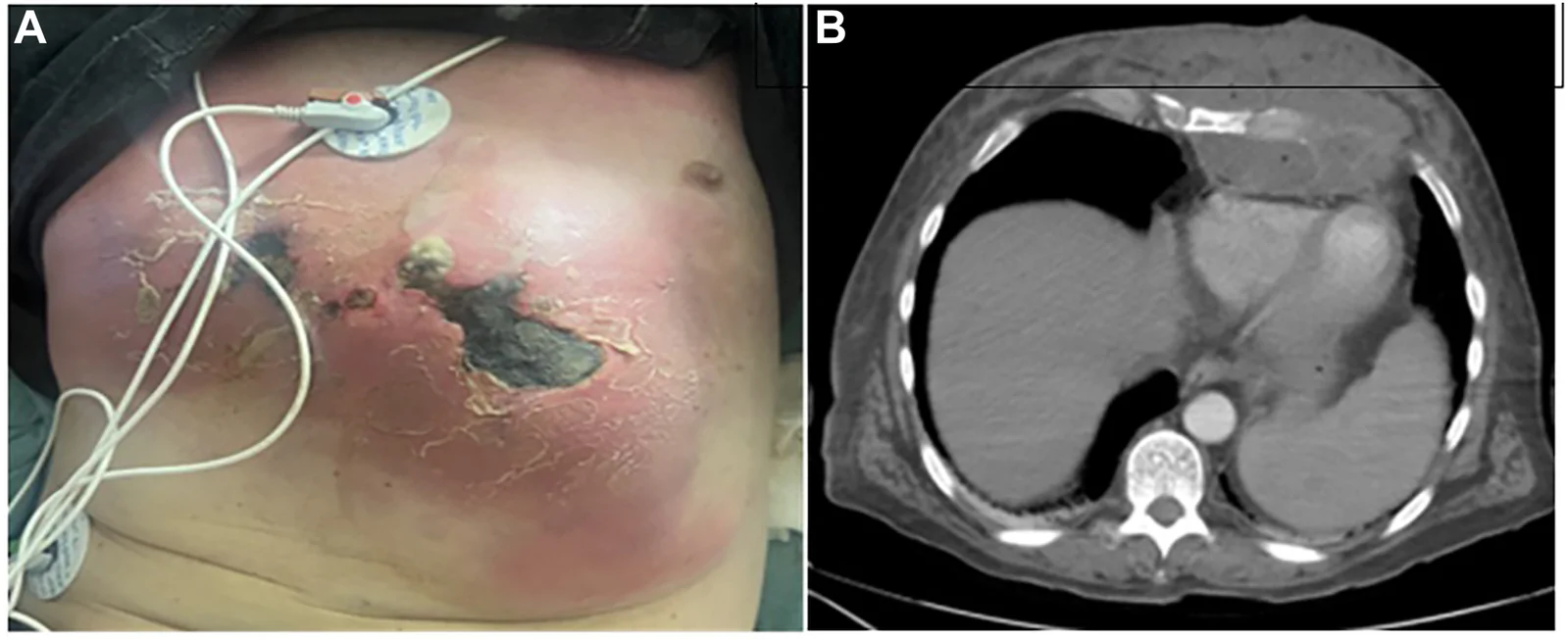
(A) Admission photograph of an extensive chest wall soft tissue infection with necrosis of skin over an open costal arch fracture. (B) Axial computed tomographic scan showing an abscess of the anterior chest wall and a soft tissue infection consistent with necrotizing soft tissue infection.

The patient was hemodynamically unstable and was taken on an emergency basis to the operating room, after resuscitation on the day of presentation. He underwent extensive debridement of necrotizing soft tissue of the anterior chest wall (Figure 2A); cultures grew group B *Streptococcus*. He was managed with broad-spectrum antibiotics, vasopressive agents, nutritional supplementation, and supportive care; multiple surgical debridements were required over 3 days. Biologic mesh of acellular dermal matrix (Strattice, AbbVie, Inc) was placed to support cadaveric skin allografts. After 2 weeks, healthy granulation tissue was present. Cadaver allografts were removed, and multiple split-thickness skin grafts (STSGs) were placed.

**Figure 2:**
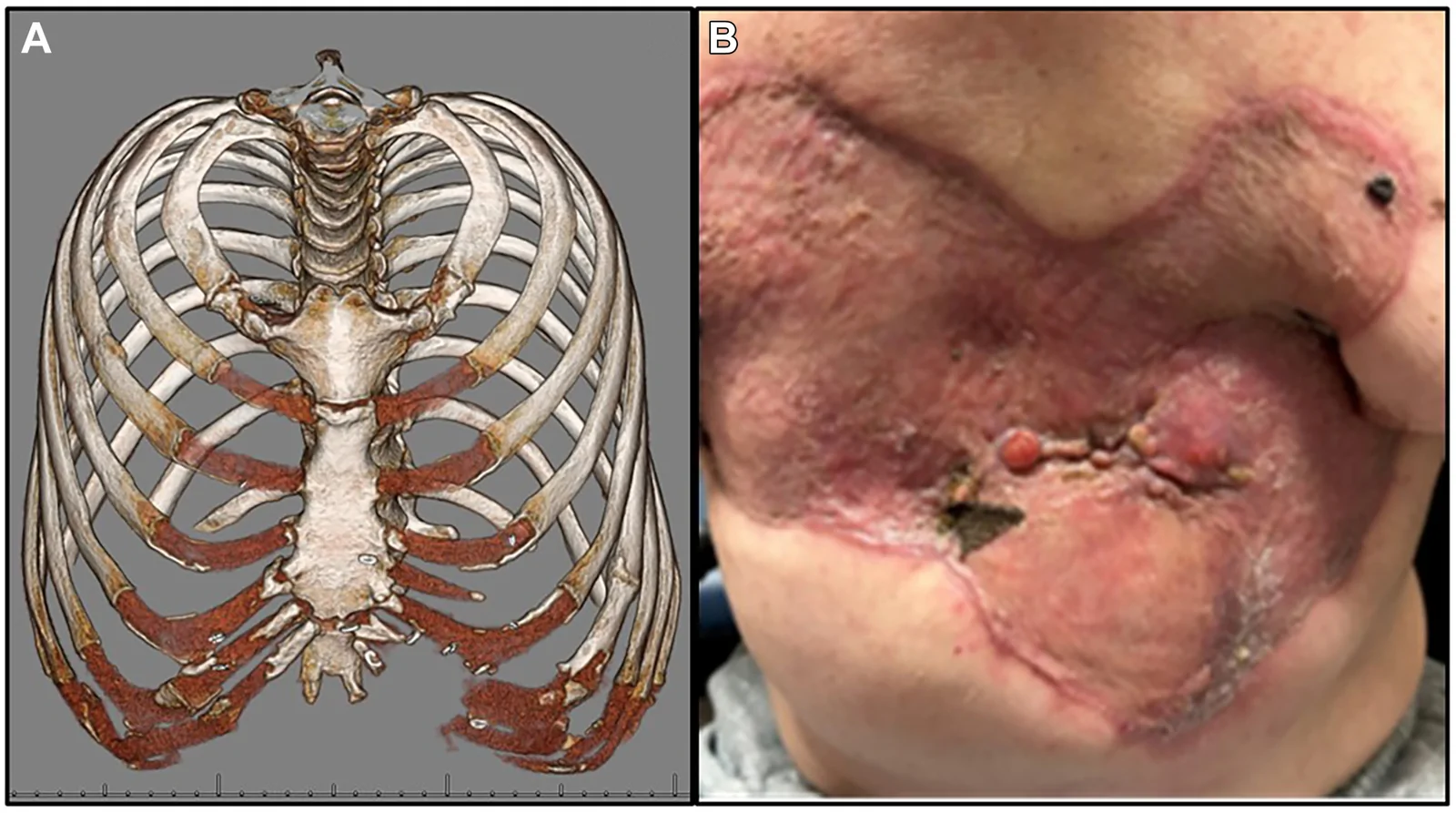
(A) Preoperative 3-dimensional reconstruction computed tomographic scan showing avulsion fractures of the left costal arch from the sternum. (B) Postoperative photograph of the patient’s chest 6 months postoperatively.

In the setting of an infected wound and an increased risk of hardware infection, polylactic acid (PLA) bars (BioBridge, Acumed, Inc) were selected for reconstruction of the costal arch (seventh and eighth costal cartilages to the sternum). An omental flap was mobilized (Figure 3A) to cover the stabilization PLA bars (Figure 3B). The flap was temporarily covered with cadaver allografts until final coverage was completed with a STSGs on postoperative day 30. A summary of the patient’s operative procedures and timeline is outlined as follows: hospital day 0, initial debridement; day 1, repeat debridement; day 3, debridement, cadaver allografts, and Strattice mesh; day 9, debridement and cadaver grafts; day 16,– STSGs; day 25, PLA bars and omental flap reconstruction; and day 30, final STSGs.

**Figure 3:**
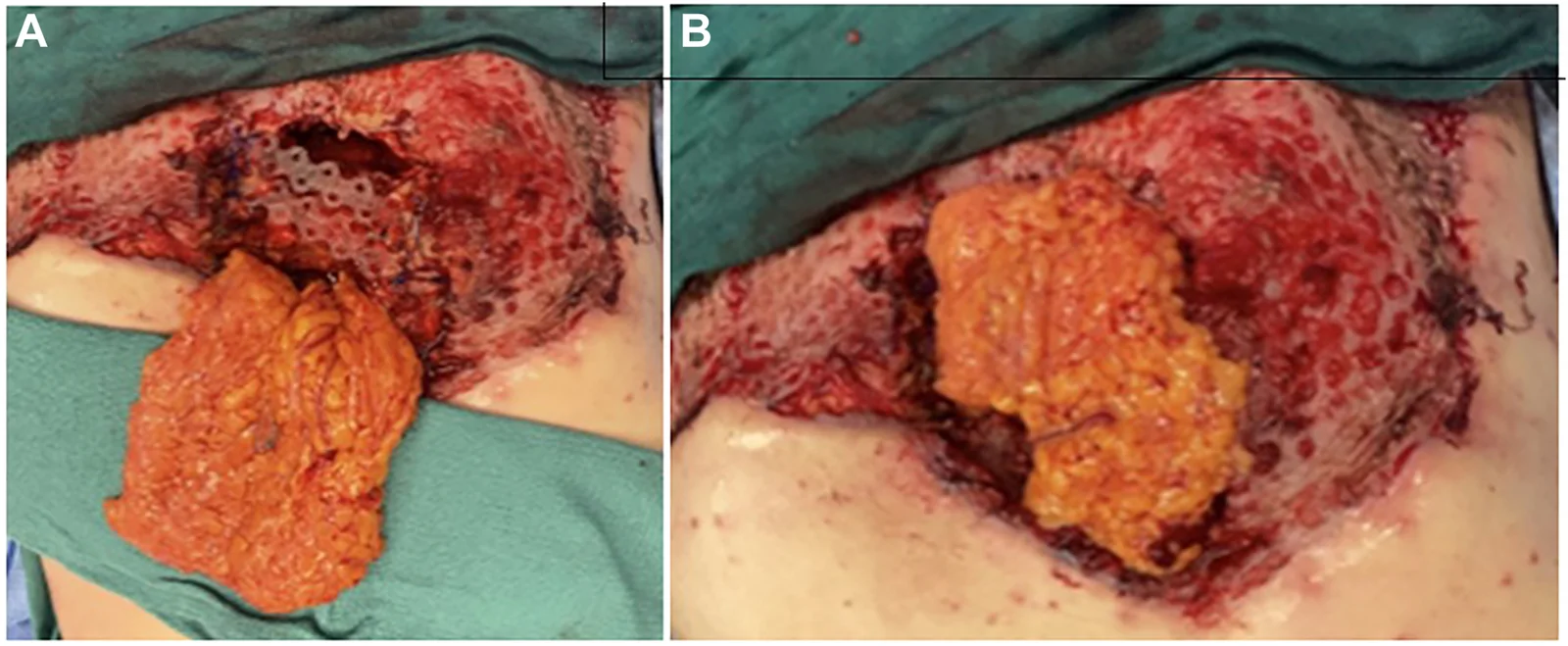
(A) Intraoperative photograph after placement of polylactic acid bars and a mobilized omental flap. (B) Intraoperative photograph after placement of an omental flap for soft tissue coverage.

The patient had multiple follow-up appointments after discharge. At his last clinic visit (6 months after discharge), he continued to have viable skin coverage, to be free of infection, and to have excellent chest wall stability (Figure 2B). A team consisting of our trauma, acute care, and thoracic surgeons (Medical College of Georgia, Augusta University, Augusta, GA) provided all of the hospital care and multiple surgical procedures.

## Comment

Delayed presentation after blunt chest wall trauma resulted in an extensive infection of the chest caused by an open fracture of the costal arch. In the setting of active infection and sepsis, delayed reconstruction was required with biomaterials and viable tissue flaps to decrease the risk of hardware contamination. Successful management of NSTI relies on aggressive debridement and infection control while planning for reconstruction. Key objectives include eradicating the infection, stabilizing the chest, and achieving viable wound coverage as early as possible.^7^ We used a staged approach: serial tissue debridements, biologic grafts, biomaterial implants for costal arch stabilization, omental flap coverage, and skin grafts.

Chest wall reconstruction requires skeletal rigidity while maintaining chest wall mechanics and providing soft tissue coverage with minimal deformity.^1^ In infected cases, biologic material is less likely to become infected after implantation. This was the reasoning for selecting PLA bars for reconstruction of the costal arch. These implants provided the required stability for the costal arch and would not be a source of continued infection. These bars reabsorb over 18 to 24 months and are excellent products for cartilaginous reconstruction. Titanium plates and screws are not well suited for the cartilaginous arch because of significant dislodgment issues and the risk of recurrent infections.^3^

The omental flap was used to fill the soft tissue defect, to cover the PLA reconstruction, and to provide a site for the STSGs. The greater omentum is a powerful tool in managing complex thoracic infections because of its rich vascular supply, immunologic properties, and significant mobilization possibilities.^8^ The omental flap was used because the other possible chest wall flaps were not available given the extensive debridements. Once the well-vascularized wound bed was established, there was no sign of infection, and the PLA bars were well covered, STSGs were placed.

The reconstructive strategy described achieved a favorable outcome for our patient, with the chest wall defect fully healed with no signs of infection or instability. Long-term prognosis depends on both the clearance of the infection and the durability of the reconstruction. Patients who survive chest wall NSTIs and undergo successful reconstruction can expect normal functional recovery of chest wall mechanics. This case shows that a staged process combining infection management with thorough surgical debridement and reconstruction can successfully treat extensive chest wall soft tissue defects from NSTI and improve survival and quality of life.
